# Negative Neurodynamic Tests Do Not Exclude Neural Dysfunction in Patients With Entrapment Neuropathies

**DOI:** 10.1016/j.apmr.2016.06.019

**Published:** 2017-03

**Authors:** Larissa T. Baselgia, David L. Bennett, Robert M. Silbiger, Annina B. Schmid

**Affiliations:** a Institute of Physiotherapy, Zurich University of Applied Sciences ZHAW, Winterthur, Switzerland; b Nuffield Department of Clinical Neurosciences, University of Oxford, Headington, UK; c Silberling Limited, Oxford, UK; d School of Health and Rehabilitation Sciences, The University of Queensland, St Lucia, QLD, Australia

**Keywords:** Carpal tunnel syndrome, Diagnosis, Nerve compression syndromes, Neurologic examination, Peripheral nerve injuries, Rehabilitation, CTS, carpal tunnel syndrome, QST, quantitative sensory testing, ULNT, upper limb neurodynamic test

## Abstract

**Objective:**

To examine differences in somatosensory phenotypes of patients with positive and negative neurodynamic tests and compare these with healthy participants.

**Design:**

Case-control study.

**Setting:**

University department.

**Participants:**

Patients with electrodiagnostically confirmed carpal tunnel syndrome (CTS) (n=53) and people without CTS (n=26) participated in this study (N=79). Patients with CTS were subgrouped according to the results of the upper limb neurodynamic tests biasing the median nerve into patients with positive or negative neurodynamic tests.

**Interventions:**

Not applicable.

**Main Outcome Measure:**

All participants underwent quantitative sensory testing in the median innervated territory of their hand.

**Results:**

Only 46% of patients with CTS had positive neurodynamic tests. No differences were identified between groups for pain thresholds (*P*>.247). However, patients with CTS had increased mechanical (*P*<.0001) and thermal detection thresholds (*P*<.0001) compared with people without CTS. Patients with negative neurodynamic tests had a more pronounced vibration detection deficit (mean, 7.43±0.59) than people without CTS (mean, 7.89±0.22; *P*=.001). Interestingly, warm detection was the only domain differentiating positive (mean, 4.03°C±2.18°C) and negative neurodynamic test groups (6.09°C±3.70°C, *P*=.032), with patients with negative neurodynamic tests demonstrating increased loss of function.

**Conclusions:**

Patients with negative neurodynamic tests seem to have a more severe dysfunction of the unmyelinated fiber population. Our findings suggest that neurodynamic tests should not be used in isolation to judge neural involvement. Rather, they should be interpreted in the context of loss of function tests of the small fiber domain.

Neurodynamic tests are frequently used in the diagnostic workup of patients with suspected entrapment neuropathies. These tests are designed to elongate the nerve bed, therefore increasing strain on neural structures.^1^ Commonly used tests include the straight leg raise or slump test for patients with radiating back or leg pain^2^ or upper limb neurodynamic tests (ULNTs) for patients with radiating neck or arm pain.^3^, ^4^ The clinometric properties of these tests are however weak,^5^, ^6^, ^7^, ^8^ which is reflected in a subgroup of patients with normative neurodynamic tests despite a clear injury to their peripheral nervous system. For instance, some patients with lumbar radiculopathy have an inconspicuous straight leg raise,^9^ and a substantial proportion of patients with carpal tunnel syndrome (CTS) have negative ULNTs despite confirmed nerve involvement.^6^, ^10^ The question arises why this discrepancy exists and whether there might be a physiological explanation for the presence of negative neurodynamic tests despite neural tissue compromise.

Whereas a potential compromise in neural function (loss of function) is commonly tested with neurologic integrity tests (sensation, muscle strength, and reflexes), neurodynamic tests have been proposed to identify alterations of mechanosensitivity in neural tissue,^1^, ^11^, ^12^ thus testing gain of function. It is therefore commonly accepted that minor nerve injuries can present with positive neurodynamic tests, even if neurologic integrity tests are normative.^13^ Given that neurodynamic tests are thought to identify gain of function (ie, heightened mechanosensitivity), we hypothesize that patients with positive neurodynamic tests are characterized by gain of function, whereas loss of function may prevail in those with negative neurodynamic tests.

Quantitative sensory testing (QST) is a well-established method that allows detailed evaluation of somatosensory phenotypes, including gain and loss of function within small and large fiber domains. Whereas some studies have used QST in patient populations with positive neurodynamic tests (eg, radiculopathy, nonspecific neck or arm pain^14^, ^15^), to our knowledge, no study has investigated whether the somatosensory phenotypes differ between patients with positive and negative neurodynamic tests. We therefore used QST to examine potential differences in somatosensory phenotypes of patients with electrodiagnostically proven CTS, who have either positive or negative neurodynamic tests, and compare their phenotypes with people without CTS.

## Methods

### Design

This study uses a cross-sectional design and includes data from the Oxford CTS cohort from May 2013 until August 2015. Data on a subgroup of this cohort have previously been published.^16^

### Participants

Fifty-three patients who met electrodiagnostic^17^ and clinical^18^ criteria for CTS were enrolled in the study. Patients were recruited through Oxford University Hospitals, local print media, and public notice boards. Patients were excluded if electrodiagnostic findings were indicative of other peripheral neuropathies (eg, cubital tunnel syndrome), if another medical condition affecting the upper extremity or neck was present (eg, tennis elbow), if a previous history of surgery or trauma to the upper limb or neck existed, or if CTS was caused by pregnancy or diabetes.

In addition, 26 volunteers without CTS, who were proportionally matched to patients with CTS according to age and sex and who had no indication of peripheral neuropathies (normative electrodiagnostic and clinical examination) or previous surgery on the upper limb or neck, were included. The study was approved by the local ethics committee, and all participants gave informed written consent prior to participating.

### Subgroups

The cohort was divided in 3 subgroups: participants without CTS, patients with CTS with positive neurodynamic tests biasing the median nerve (ULNTs 1 and/or 2a),^1^ and patients with CTS with negative ULNTs 1 and 2a. Because clinically the test outcomes of ULNTs 1 and 2a are not necessarily mutually inclusive in patients with CTS, both tests were included to assure the identification of any mechanosensitivity of the median nerve. The ULNTs were performed by a single experienced musculoskeletal physiotherapist in a standardized way.^19^ Patients were positioned in a supine position with the hand of the untested side resting on the participant's abdomen. The ULNT 1 sequence involved shoulder girdle fixation, shoulder abduction, wrist extension, supination, shoulder external rotation, followed by elbow extension. The ULNT 2a included shoulder girdle depression, elbow extension, shoulder external rotation, supination and wrist extension, followed by shoulder abduction. All components were performed to the end of range or until symptoms were provoked. Patients were instructed to report the onset of any sensations (eg, stretch, tingling or pain anywhere in the upper quadrant or neck). ULNTs were rated as positive if the patients' current symptoms could be at least partially reproduced and if a structural differentiation (sensitizing movements at a site distant to the symptoms) was indicative of neural involvement^1^, ^19^ (eg, shoulder elevation relieves finger symptoms during ULNT 2a). This standardized performance is in accordance with recommended criteria for the execution and interpretation of ULNTs^20^ and has shown moderate intertester reliability.^19^ Participants were familiarized with this test procedure on the nonexperimental arm before testing the experimental side.

### Questionnaires

All participants completed the Pain Anxiety Symptom Scale^21^ and the Pain Catastrophizing Scale rumination, magnification, and helplessness subscales^22^ to examine a potential difference in psychological factors between the 3 groups. Patients with CTS also completed the Boston Carpal Tunnel Questionnaire,^23^ the Neuropathic Pain Symptom Inventory,^24^ and the Leeds Assessment of Neuropathic Symptoms Scale.^25^

### Quantitative Sensory Testing

QST was performed according to the standardized protocol of the German Research Network on Neuropathic Pain by a trained investigator.^26^ This protocol is designed to comprehensively examine loss and gain of function in different fiber domains, including thick myelinated, thin myelinated, and unmyelinated fibers. For thermal testing, a Thermotesta (thermode size: 25×50mm) was used to measure the cold detection threshold and warm detection threshold and the cold pain threshold and heat pain threshold. We also recorded paradoxical heat sensations during thermal sensory limen testing. The mechanical detection threshold was measured with Von Frey hairs,b and the mechanical pain threshold was measured with weighted PinPrick stimulators.c The pressure pain threshold was determined with a manual algometer,d and the vibration detection threshold was determined with a Rydel Seiffer tuning fork.e Mechanical pain sensitivity was determined with a numerical pain rating scale (0–100) during 5 sets of 7 pseudorandom pin-prick stimulations. Intermingled with these pin-prick stimulations were 5 sets of 3 light-touch stimulations with a cotton wisp, a cotton wool tip, and a standardized brush (Sense-laba). The wind up ratio was determined as the mean numerical pain rating of 5 trains of 10 pin-prick stimulations divided by the mean rating of 5 single stimuli. More details on this standardized QST protocol and the exact calculation of the values can be found elsewhere.^27^

All subjects were familiarized with the QST procedure on the dorsum of the hand before the actual measurements started. QST data were collected in the median nerve territory on the palmar side of the index finger. The pressure pain threshold was recorded over the thenar eminence, and the vibration detection threshold was tested over the palmar side of the distal end of the second metacarpal. In patients, the more affected hand was taken as the test site. Because QST parameters do not differ between sides, we standardized the QST measurements to the nondominant hand in those without CTS.^27^ To achieve normally distributed data, all QST parameters except for the cold and heat pain thresholds and vibration detection thresholds were log-transformed^28^, ^29^ and expressed as *z* scores.^27^ A small constant of 0.1 was added to the mechanical pain sensitivity to avoid loss of zero rating values.^26^ To allow group comparison, *z* scores were calculated as *z*=(value of the participant−mean value of healthy controls)/SD of healthy controls. Positive *z* scores therefore represent gain of function, whereas negative values indicate loss of function.^27^

### Statistical analysis

IBM SPSS Statistics 22f was used for statistical analysis. The *z* scores of the QST data and the scores from the Pain Catastrophizing Scale, Pain Anxiety Symptom Scale, and Depression, Anxiety and Positive Outlook Scale were compared among the 3 groups with 1-way analyses of variance followed by Tukey post hoc analysis. The Boston questionnaire, Neuropathic Pain Symptom Inventory, and Leeds Assessment of Neuropathic Symptoms Scale were compared among the 2 groups of patients with CTS using independent *t* tests. Sample size calculation based on previously published data of our cohort^16^ revealed that at least 24 participants are needed per group to identify significant differences in detection thresholds among groups with 80% power and significance set at *P*=.05 (effect sizes, .36<Cohen's f>.56).g This sample size also allows the detection of a 20% difference in pressure pain thresholds between groups, which is more sensitive than the reported clinically relevant difference of 36%.^27^

## Results

### Demographic variables and questionnaires

The study overall included 79 participants: people without CTS (n=26, 18 women), patients with CTS with negative ULNTs (n=29, 14 women), and patients with CTS with positive ULNTs (n=24, 18 women). Within the positive ULNT subgroup, 41.6% of patients had both positive ULNT 1 and 2a (n=10), 37.5% had only a positive ULNT 2a (n=9), and 20.8% had only a positive ULNT 1 (n=5).

Age (*P*=.134), height (*P*=.105), weight (*P*=.071), and sex distributions (*P*=.176) were comparable between groups (table 1). For psychological variables, no differences were detected between groups for all scales (*P*>.08) except for the Pain Anxiety Symptom Scale and Depression and the positive outlook subscale of the Anxiety and Positive Outlook Scale, in which patients with CTS were significantly different from participants without CTS independent of the ULNT subgroup (*P*<.029) (see table 1). The duration of symptoms in the patient groups (*P*=.311), the electrodiagnostic severity graded with the Bland scale (*P*=.954),^30^ the scores of the Boston questionnaire for both symptoms and function (*P*>.358), and the measures of neuropathic pain (Neuropathic Pain Symptom Inventory: *P*=.904, Leeds Assessment of Neuropathic Symptoms Scale: *P*=.726) were comparable between the positive and negative ULNT groups (see table 1).

### Somatosensory phenotype

**Fig 1:**
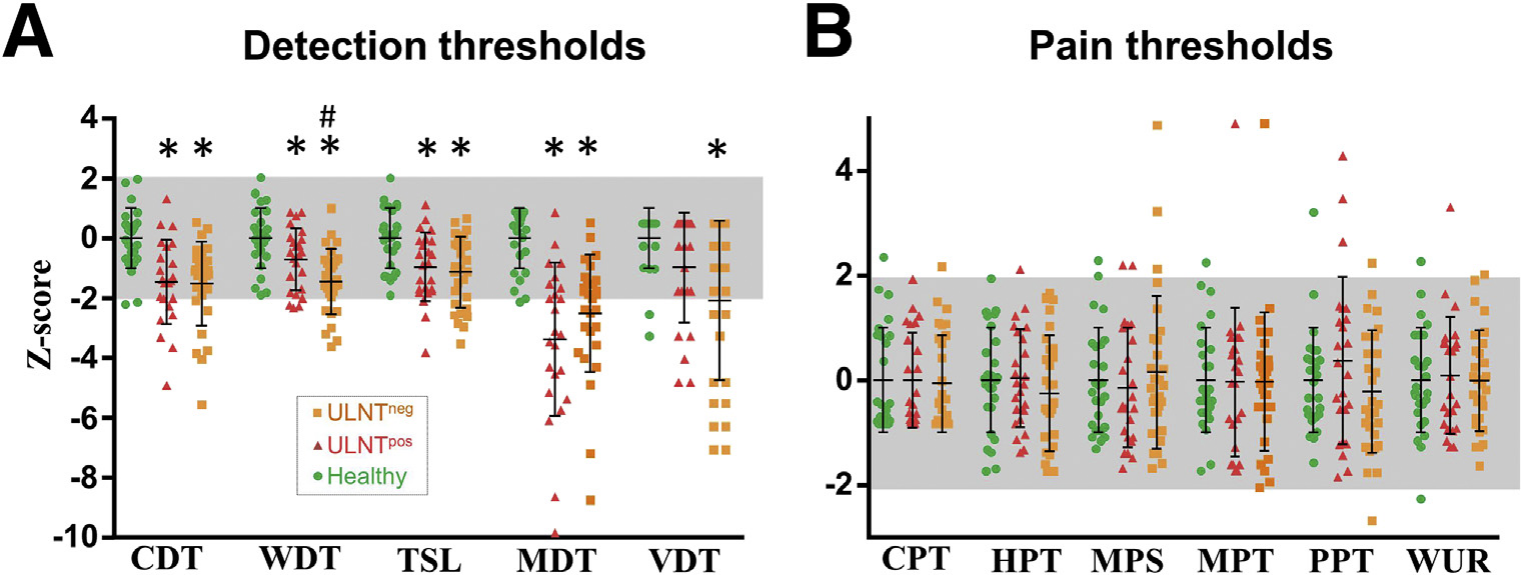
Somatosensory phenotype. The mean *z* score sensory profiles are shown of participants without CTS (*green*), patients with ULNT^pos^ (*red*), and patients with ULNT^neg^ (*orange*). (A) QST domains reflecting detection thresholds. (B) Pain thresholds. ^∗^Significance compared with participants without CTS. ^#^Significance between CTS groups. Error bars represent SDs. Abbreviations: TSL, thermal sensory limen; ULNT^neg^, negative neurodynamic tests; ULNT^pos^, positive neurodynamic tests; WUR, wind up ratio.

For detection thresholds, we found a significant difference between groups for the cold and warm detection thresholds and thermal sensory limen testing (all *P*<.0001) and the vibration and mechanical detection thresholds (*P*=.001 and *P*<.0001 respectively) (table 2 and fig 1A). Post hoc analysis demonstrated that both the positive and negative ULNT groups had significantly more loss of function in the cold and warm detection thresholds, sensory limen testing, and mechanical detection threshold than participants without CTS (all *P*<.047). For the vibration detection thresholds, patients with negative ULNTs had a reduced vibration detection sense compared with participants without CTS (*P*=.001), but no differences were found between participants without CTS and patients with positive ULNTs (*P*=.193) or between patient groups (*P*=.119). The only significant difference between the negative and positive ULNT groups was for the warm detection thresholds (*P*=.032), which revealed a more pronounced deficit in patients with negative ULNTs, indicating increased loss of unmyelinated fiber function.

We did not identify any significant differences between groups for all pain thresholds (cold, heat, mechanical, and pressure pain thresholds all *P*>.247) (fig 1B) and mechanical pain sensitivity (*P*=.697). One patient in each ULNT group presented with a paradoxical heat sensation, whereas no participant had a dynamic mechanical allodynia.

## Discussion

The findings of this study suggest that >54% of patients with CTS have negative neurodynamic tests despite a clear median nerve dysfunction as proven with electrodiagnostic tests. Interestingly, patients with negative ULNTs have a somatosensory phenotype, indicating a more pronounced impairment of warm detection sense than patients with positive ULNTs. Our findings suggest that neurodynamic tests in isolation are not sufficient to determine neural involvement. Indeed, patients with negative ULNTs seem to have a more pronounced dysfunction of the small unmyelinated nerve fiber population than patients with positive ULNTs.

Our finding that a significant proportion of patients with proven nerve involvement have negative neurodynamic tests is in line with previous studies. For instance, approximately 58% of patients with lumbosacral radiculopathy have a negative straight leg raise,^8^ and for CTS, the percentage of positive ULNTs varies from 18% to 72%.^5^, ^6^, ^7^ This variation in prevalence values among the different studies is most likely explained by the different performances of ULNTs and the varying criteria for a positive test. For instance, some studies used structural differentiation as an essential criterion to rate a neurodynamic test as positive,^5^ whereas structural differentiation was not required for a positive test outcome in other studies.^7^ We strictly adhered to the recommended criteria for a standardized performance and interpretation of ULNTs,^20^ which includes structural differentiation as an essential test criterion.

Not surprisingly, both ULNTs biasing the median nerve were positive only in approximately 42% of our patients. Originally, the ULNT 1 was developed to increase the strain on the brachial plexus,^3^ whereas ULNT 2a includes a prolonged period of strain on the distal median nerve. This may explain why positive neurodynamic tests were more prevalent for the ULNT 2a than the ULNT 1. Following the principles of sequencing,^31^ it could be argued that further test adjustment, such that the wrist is moved into dorsal extension first, would increase the likelihood of a positive neurodynamic test in patients with CTS. A recent cadaver study has however demonstrated that changing the order of movement during neurodynamic testing does not affect the extent of strain of the median nerve at the distal forearm.^32^

In accordance with previous studies,^16^, ^33^, ^34^, ^35^ patients with CTS had a somatosensory phenotype indicating loss of function compared with participants without CTS. This was apparent for both thermal and mechanical stimuli, which indicates a dysfunction affecting both the large and small fiber spectrum. Intriguingly, the extent of loss of function was comparable between patients with negative and positive neurodynamic tests apart from a more pronounced deficit in warm detection in the negative ULNT group. This seems counterintuitive because it suggests a more severe nerve dysfunction of unmyelinated fibers in those with negative neurodynamic tests. Small unmyelinated C fibers mediate warm sensations,^26^ but a subgroup also has nociceptive function. It has previously been shown that passive limb extension after experimental nerve injury leads to an afferent barrage, including the C fiber population.^36^ Recent studies suggest that entrapment neuropathies can affect small fiber function^34^, ^37^ and may even lead to their structural degeneration.^16^ Potentially, a more severe dysfunction of these small fibers may result in their decreased firing on neurodynamic testing. In addition, the small (mostly unmyelinated) fiber population also innervates the epineurium as nervi nervorum.^38^ It has previously been argued that neural mechanosensitivity may in part be attributed to a hyperexcitability of these intrinsic nociceptive afferents.^39^ It could be speculated that a similar loss of small fiber function as identified in target tissue may also affect the nervi nervorum, therefore leading to a reduced response during neurodynamic testing.

Future studies will have to examine whether our findings of a more pronounced dysfunction of the unmyelinated fibers in patients with negative neurodynamic tests also applies to conditions affecting the lower extremity. There is some indication that the response to the straight leg raise is diminished in patients with severe diabetic neuropathy.^40^ However, neuropathy severity was based on vibration detection thresholds, which are mediated by large myelinated fibers, and small fiber function was not examined. In our study, vibration detection was significantly impaired in patients with negative ULNTs. It can however be debated whether the difference of 0.46 out of 8 on the tuning fork scale is of clinical relevance.^26^ In addition, the opposing trend in mechanical detection thresholds and comparable electrodiagnostic test severity suggests that the function of the large fiber population is not the predominant feature that differentiates patients with CTS with and without positive neurodynamic tests.

Whereas we confirmed our hypothesis that patients with negative neurodynamic tests are characterized by predominant loss of function, our second hypothesis that positive neurodynamic tests are associated with increased gain of function was not substantiated. In contrast with previous studies in patients with CTS,^41^, ^42^, ^43^, ^44^ our cohort was not characterized by gain of function in any of the QST parameters. It has previously been shown that patients with CTS have increased mechanosensitivity when directly testing over the median nerve trunk,^42^ presumably mediated by nervi nervorum.^39^ Future studies should include QST over the nerve trunks to determine whether positive neurodynamic tests are associated with elevated nerve trunk sensitivity rather than its reflection in target tissues.

It could be argued that the slightly (albeit statistically insignificant) higher proportion of men in the negative ULNT group compared with the positive ULNT group (*P*=.097) may have influenced our findings, especially for pain thresholds, which are affected by sex.^26^ Repeating the analysis with sex as a covariate did however not change our results (data not shown). This together with the comparability of the subgroups in regard to other demographic, clinical, and psychological variables makes it unlikely that our results are biased by confounding variables.

### Study limitations

The only parameter that differentiated the negative and positive ULNT groups was a deficit in warm detection. Because the QST battery involves 13 parameters, the likelihood of a false-positive result increases. Each QST modality does however test highly distinct fiber types (eg, thick myelinated vs thin myelinated vs unmyelinated),^26^ which are not necessarily affected in the same way in patients with entrapment neuropathies.^14^, ^16^, ^35^ It is therefore commonly accepted that analysis of QST parameters does not require correction for multiple testing.^45^ Future studies are however required to confirm our findings both in CTS and in other peripheral neuropathies.

### Clinical implications

Because electrodiagnostic or imaging results are often not available, practitioners commonly rely on clinical tests (eg, neurodynamic or neurologic examination) when diagnosing patients with suspected entrapment neuropathies. The presence of a negative neurodynamic test is commonly interpreted as diminishing the likelihood for neural involvement. Our findings however suggest the opposite, namely that negative neurodynamic tests may reflect the presence of a more pronounced dysfunction, specifically of unmyelinated fibers. Recent studies demonstrated that small fiber deficits may precede large fiber dysfunction in patients with CTS.^16^, ^34^ This together with our results suggests that testing of small fibers is warranted before a meaningful interpretation of neurodynamic tests is possible. Loss of function of unmyelinated fibers is clinically identified using the warm detection threshold.^27^ If quantitative thermal testing is unavailable, clinicians may use cold and warm metallic objects to identify potential differences in thermal thresholds.^46^, ^47^ Future studies are however needed to determine the validity of simple but cost-effective bedside tests.

## Conclusions

A substantial proportion of patients with CTS have negative neurodynamic tests despite a clear nerve involvement as determined with electrodiagnostic studies. Interestingly, patients with negative neurodynamic tests were characterized by a more substantial loss of function in warm detection than patients with positive neurodynamic test responses. This suggests a more pronounced dysfunction of small unmyelinated fibers in patients with negative neurodynamic tests. Therefore, when diagnosing neural involvement clinically, neurodynamic test outcomes should not be interpreted in isolation, but in the context of a sound clinical reasoning specifically including neurologic integrity tests of the small unmyelinated fiber population.

## Suppliers

a. Somedic.
b. Optihair; MARSTOCK nervtest.
c. PinPrick stimulators; MRC Systems.
d. Manual algometer; Wagner Instruments.
e. Rydel Seiffer tuning fork; US Neurologicals, Washington.
f. IBM SPSS Statistics 22; IBM.
g. G*Power; Universität Düsseldorf.

## Figures and Tables

**Table 1:** Demographic and clinical variables of included participants

Variable	ULNT^pos^ Group	ULNT^neg^ Group	Participants Without CTS	*P*
Age (y)	54.1±13.9	59.2±13.9	51.0±17.3	.134
Sex (f/m)	18/6	14/15	18/8	.176
Height (cm)	163.5±11.6	169.6±10.9	168.4±9.9	.105
Weight (kg)	73.8±11.5	81.4±18.9	72.1±15.3	.071
BMI (kg/m^2^)	27.1±7.5	27.8±7.6	24.5±6.1	.193
Symptom duration (m)	55.0±68.1	76.1±81.9		.311
PCS	9.5±10.0	14.1±12.3	7.9±8.9	.080
DAPOS				
Depression	6.23±3.34	7.00±4.50	6.77±2.93	.746
Anxiety	4.08±2.84	4.38±2.77	3.73±1.22	.608
Outlook	9.73±4.77∗	9.76±4.62∗	12.38±2.26	.029
PASS	21.5±18.8∗	19.6±17.7∗	8.4±11.6	.011
EDT grade				.954
Very mild	3	5		
Mild	6	6		
Moderate	8	9		
Severe	4	5		
Very severe	3	4		
Boston questionnaire				
Symptoms	2.60±0.7	2.71±0.6		.544
Function	1.98±0.6	2.17±0.82		.358
NPSI	4.9±8.2	4.64± 7.7		.904
s-LANSS	8.88±5.97	8.28±6.30		.726

NOTE. Values are mean ± SD or as otherwise indicated.

Abbreviations: BMI, body mass index; DAPOS, Depression, Anxiety and Positive Outlook Scale; EDT, electrodiagnostic tests; f, female; m, male; NPSI, Neuropathic Pain Symptom Inventory; PASS, Pain Anxiety Symptoms Scale; PCS, Pain Catastrophizing Scale; s-LANSS, self-version of the Leeds Assessment of Neuropathic Symptoms Scale; ULNT^neg^, negative upper limb neurodynamic test; ULNT^pos^, positive upper limb neurodynamic tests.

∗ Indicates statistically significant differences compared to participants without CTS.

**Table 2:** QST parameters

QST Parameter	ULNT^pos^ Group	ULNT^neg^ Group	Participants Without CTS	*P* (ANOVA)
CDT (deg Celsius)	4.01±2.64∗	4.29±3.26∗	2.17±0.92	<.0001
WDT (deg Celsius)	4.03±2.18∗	6.09±3.70∗†	2.84±1.53	<.0001
TSL (deg Celsius)	8.89±6.56∗	10.07±6.08∗	5.13±2.72	<.0001
MDT (mN)	7.67±18.59∗	3.41±8.78∗	0.36±0.25	<.0001
VDT (×/8)	7.67±0.40	7.43±0.59∗	7.89±0.22	.001
CPT (deg Celsius)	9.89±6.42	9.41±6.57	10.02±7.18	.954
HPT (deg Celsius)	43.35±3.53	44.44±4.17	43.46±3.84	.526
MPT (mN)	166.44±111.61	162.46±112.18	150.87±79.72	.994
MPS (rating 0–100)	0.68±0.85	2.00±6.29	0.71±0.71	.697
WUR	2.43±1.96	2.17±1.19	2.10±1.15	.925
PPT (kPa)	330.17±125.23	375.40±118.26	347.19±82.28	.247

NOTE. Data are presented as mean ± SD for untransformed data (CPT, HPT, and VDT), retransformed mean for log-transformed data. The *P* values reflect statistics done on *z* scores.

Abbreviations: ANOVA, analysis of variance; CDT, cold detection threshold; CPT, cold pain threshold; HPT, heat pain threshold; MDT, mechanical detection threshold; MPS, mechanical pain sensitivity; MPT, mechanical pain threshold; PPT, pressure pain threshold; QST, quantitative sensory testing; TSL, thermal sensory limen; ULNT^neg^, negative upper limb neurodynamic test; ULNT^pos^, positive upper limb neurodynamic tests; VDT, vibration detection threshold; WDT, warm detection threshold; WUR, wind up ratio.

∗ Significant difference compared with participants without CTS.

† Significant difference compared with the ULNT^pos^ group.
